# Supervised and Unsupervised Self-Testing for HIV in High- and Low-Risk Populations: A Systematic Review

**DOI:** 10.1371/journal.pmed.1001414

**Published:** 2013-04-02

**Authors:** Nitika Pant Pai, Jigyasa Sharma, Sushmita Shivkumar, Sabrina Pillay, Caroline Vadnais, Lawrence Joseph, Keertan Dheda, Rosanna W. Peeling

**Affiliations:** 1 Division of Clinical Epidemiology, McGill University Health Centre, Department of Medicine, McGill University, Montreal, Canada; 2 Department of Epidemiology, Biostatistics and Occupational Health, McGill University, Montreal, Canada; 3 Lung Infection and Immunity Unit, Division of Pulmonology and UCT Lung Institute, Department of Medicine and Institute of Infectious Diseases and Molecular Medicine, University of Cape Town, Cape Town, South Africa; 4 London School of Hygiene and Tropical Medicine, London, United Kingdom; University of California, San Francisco, United States of America

## Abstract

**Background:**

Stigma, discrimination, lack of privacy, and long waiting times partly explain why six out of ten individuals living with HIV do not access facility-based testing. By circumventing these barriers, self-testing offers potential for more people to know their sero-status. Recent approval of an in-home HIV self test in the US has sparked self-testing initiatives, yet data on acceptability, feasibility, and linkages to care are limited. We systematically reviewed evidence on supervised (self-testing and counselling aided by a health care professional) and unsupervised (performed by self-tester with access to phone/internet counselling) self-testing strategies.

**Methods and Findings:**

Seven databases (Medline [via PubMed], Biosis, PsycINFO, Cinahl, African Medicus, LILACS, and EMBASE) and conference abstracts of six major HIV/sexually transmitted infections conferences were searched from 1st January 2000–30th October 2012. 1,221 citations were identified and 21 studies included for review. Seven studies evaluated an unsupervised strategy and 14 evaluated a supervised strategy. For both strategies, data on acceptability (range: 74%–96%), preference (range: 61%–91%), and partner self-testing (range: 80%–97%) were high. A high specificity (range: 99.8%–100%) was observed for both strategies, while a lower sensitivity was reported in the unsupervised (range: 92.9%–100%; one study) versus supervised (range: 97.4%–97.9%; three studies) strategy. Regarding feasibility of linkage to counselling and care, 96% (*n* = 102/106) of individuals testing positive for HIV stated they would seek post-test counselling (unsupervised strategy, one study). No extreme adverse events were noted. The majority of data (*n* = 11,019/12,402 individuals, 89%) were from high-income settings and 71% (*n* = 15/21) of studies were cross-sectional in design, thus limiting our analysis.

**Conclusions:**

Both supervised and unsupervised testing strategies were highly acceptable, preferred, and more likely to result in partner self-testing. However, no studies evaluated post-test linkage with counselling and treatment outcomes and reporting quality was poor. Thus, controlled trials of high quality from diverse settings are warranted to confirm and extend these findings.

*Please see later in the article for the Editors' Summary*

## Abstract 2

By systematically reviewing the literature, Nitika Pant Pai and colleagues assess the evidence base for HIV self tests both with and without supervision.

## Editors' Summary

**Background:**

About 34 million people (most living in resource-limited countries) are currently infected with HIV, the virus that causes AIDS, and about 2.5 million people become infected with HIV every year. HIV is usually transmitted through unprotected sex with an infected partner. HIV infection is usually diagnosed by looking for antibodies to HIV in blood or saliva. Early during infection, the immune system responds to HIV by beginning to make antibodies that recognize the virus and target it for destruction. “Seroconversion”—the presence of detectable amounts of antibody in the blood or saliva—usually takes 6–12 weeks. Rapid antibody-based tests, which do not require laboratory facilities, can provide a preliminary result about an individual's HIV status from a simple oral swab or finger stick sample within 20 minutes. However preliminary rapid positive results have to be confirmed in a laboratory, which may take a few days or weeks. If positive, HIV infection can be controlled but not cured by taking a daily cocktail of powerful antiretroviral drugs throughout life.

**Why Was This Study Done?:**

To reduce the spread of HIV, it is essential that HIV-positive individuals get tested, change behaviors avoid transmitting the virus to other people by, for example, always using a condom during sex, and if positive get on to treatment that is available worldwide. Treatment also reduces transmission of virus to the partner and controls the virus in the community. However, only half the people currently living with HIV know their HIV status, a state of affairs that increases the possibility of further HIV transmission to their partners and children. HIV positive individuals are diagnosed late with advanced HIV infection that costs health care services. Although health care facility-based HIV testing has been available for decades, people worry about stigma, visibility, and social discrimination. They also dislike the lack of privacy and do not like having to wait for their test results. Self-testing (i.e., self-test conduct and interpretation) might alleviate some of these barriers to testing by allowing individuals to determine their HIV status in the privacy of their home and could, therefore, increase the number of individuals aware of their HIV status. This could possibly reduce transmission and, through seeking linkages to care, bring HIV under control in communities. In some communities and countries, stigma of HIV prevents people from taking action about their HIV status. Indeed, an oral (saliva-based) HIV self-test kit is now available in the US. But how acceptable, feasible, and accurate is self-testing by lay people, and will people who find themselves self-test positive seek counseling and treatment? In this systematic review (a study that uses pre-defined criteria to identify all the research on a given topic), the researchers examine these issues by analyzing data from studies that have evaluated supervised self-testing (self-testing and counseling aided by a health-care professional) and unsupervised self-testing (self-testing performed without any help but with counseling available by phone or internet).

**What Did the Researchers Do and Find?:**

The researchers identified 21 eligible studies, two-thirds of which evaluated oral self-testing and a third of which evaluated blood-based self-testing. Seven studies evaluated an unsupervised self-testing strategy and 14 evaluated a supervised strategy. Most of the data (89%) came from studies undertaken in high-income settings. The study populations varied from those at high risk of HIV infection to low-risk general populations. Across the studies, acceptability (defined as the number of people who actually self-tested divided by the number who consented to self-test) ranged from 74% to 96%. With both strategies, the specificity of self-testing (the chance of an HIV-negative person receiving a negative test result is true negative) was high but the sensitivity of self-testing (the chance of an HIV-positive person receiving a positive test result is indeed a true positive) was higher for supervised than for unsupervised testing. The researchers also found evidence that people preferred self-testing to facility-based testing and oral self-testing to blood-based self testing and, in one study, 96% of participants who self-tested positive sought post-testing counseling.

**What Do These Findings Mean?:**

These findings provide new but limited information about the feasibility, acceptability, and accuracy of HIV self-testing. They suggest that it is feasible to implement both supervised and unsupervised self-testing, that both strategies are preferred to facility-based testing, but that the accuracy of self-testing is variable. However, most of the evidence considered by the researchers came from high-income countries and from observational studies of varying quality, and data on whether people self-testing positive sought post-testing counseling (linkage to care) were only available from one evaluation of unsupervised self-testing in the US. Consequently, although these findings suggest that self-testing could engage individuals in finding our their HIV status and thereby help modify behavior thus, reduce HIV transmission in the community, by increasing the proportion of people living with HIV who know their HIV status. The researchers suggested that more data from diverse settings and preferably from controlled randomized trials must be collected before any initiatives for global scale-up of self-testing for HIV infection are implemented.

**Additional Information:**

Please access these Web sites via the online version of this summary at http://dx.doi.org/10.1371/journal.pmed.1001414.

- Information is available from the US National Institute of Allergy and Infectious Diseases on HIV infection and AIDS
- NAM/aidsmap provides basic information about HIV/AIDS and summaries of recent research findings on HIV care and treatment
- Information is available from Avert, an international AIDS charity on many aspects of HIV/AIDS, including information on HIV testing, and on HIV transmission and testing (in English and Spanish)
- The UK National Health Service Choices website provides information about all aspects of HIV and AIDS; a “behind the headlines” article provides details about the 2012 US approval for an over-the-counter HIV home-use test
- The 2012 World AIDS Day Report provides information about the percentage of people living with HIV who are aware of their HIV status in various African countries, as well as up-to-date information about the AIDS epidemic
- Patient stories about living with HIV/AIDS are available through Avert; the nonprofit website Healthtalkonline also provides personal stories about living with HIV, including stories about getting a diagnosis

## Introduction

On July 3, 2012 the US Food and Drug Administration (FDA) approved an oral point-of-care (POC) HIV self test, OraQuick, for over-the-counter (OTC) sale on the basis of a positive recommendation from their Blood Product Advisory Committee (FDA-BPAC) [1]. This decision, the first for a self test for an infectious disease, is an important step towards normalizing the process of receiving an HIV diagnosis, currently beset with stigma and discrimination. The FDA approved the oral HIV test even though it is less sensitive than a blood test because of its potential to allow more people to know their sero-status and thus potentially avert thousands of cases of HIV transmission. Although this approval has paved the way for a self-testing paradigm complementary to facility-based testing, evidence is needed to understand whether self-testing will lead to more people knowing their HIV status and whether self-testing can be implemented, and operationalized as a strategy in global settings.

If HIV self-testing is to realize its promise of increasing the number of at-risk individuals knowing their sero-status evidence is needed to demonstrate that self-testing strategies can be an acceptable or preferred mode of HIV testing. Crucially, it is important to demonstrate that individuals who are self-testing can be given reasonable assurance of test accuracy, especially for populations with varying backgrounds and literacy levels. Furthermore, evidence on whether self-testing will offer a private, confidential alternative to facility-based HIV testing with a safe conduit to care and treatment is needed to improve outcomes for both individuals and at-risk populations.

If self-testing is proven to help increase knowledge of sero-status in those individuals that do not seek facility-based testing and improves linkages to care and treatment rates in the community, then it will stand to impact control HIV at the population level. However, as of 2013, that vision remains unrealized. Facility-based HIV testing strategies (voluntary testing and counselling, provider-initiated and conventional client-initiated testing and counselling) have been in place for decades [2]. However, stigma and discrimination faced in these settings remain key barriers to testing [3]. Additional barriers include fear of visibility, fear of lack of confidentiality of a positive test result, a lack of privacy, and increased waiting time to obtain a test result [4]. It is no surprise therefore that about six in ten individuals living with HIV infection remain untested globally and as a consequence, are unaware of their HIV sero-status [5].

In this context, self-testing offers some promise in alleviating or eliminating these barriers. It provides individuals with the option of knowing their HIV status in the privacy of their homes and therefore has the potential to negate the effects of stigma, and reduce perceived discrimination. Although self-testing has the potential to help increase the numbers of individuals aware of their HIV sero-status, this strategy has been beset with concerns and challenges about its success [6]–[8]. This is also partly attributed to a lack of evidence on self-testing behaviors, and effective linkage initiation to treatment and counselling after self-testing. To our knowledge, only one published narrative review has evaluated the potential role of self-testing in health care worker populations [2]. A related meta-analysis compared the diagnostic accuracy of oral versus finger-stick antibody POC tests to demonstrate that oral POC tests could play a role in future self-testing initiatives [9]. Another systematic review on home-based rapid testing initiatives where people are encouraged to get tested and counselled by health care workers in home visits (thus different from self-testing) alluded to the potential of offering self-testing to individuals in resource constrained settings [10]. With a view to fill a knowledge gap in understanding supervised and unsupervised self-testing strategies that are being evaluated globally, and to guide their effective implementation in high-income and resource constrained settings, we performed a systematic review.

## Methods

We systematically reviewed the literature to evaluate the current evidence on two common strategies: (a) supervised and (b) unsupervised self-testing strategies in high- and low-risk populations worldwide. A supervised strategy (self-testing and counselling processes) was always aided by a health care professional (HCP). An unsupervised strategy was performed by a self-tester without any help, but with counselling and linkage to care offered off-site (e.g., over the phone) by a HCP. Our specific objectives included documentation of all outcomes from implementation research associated with self-testing and counselling strategies (acceptability, accuracy, feasibility, cost, and counselling preferences). Further, data on challenges, concerns, and barriers documented from qualitative research or mixed methods studies were also synthesized.

This review was reported following PRISMA guidelines.

### Search Strategy and Identification of Studies

For the period of January 1, 2000–October 30, 2012, we searched seven electronic databases (Medline [via PubMed], Biosis, PsycINFO, Cinahl, African Medicus, LILACS, and EMBASE) and abstracts from six major HIV/sexually transmitted infections (STIs) conferences (Canadian Association of HIV Research [CAHR], International Society for Sexually Transmitted Diseases Research [ISSTDR], International AIDS Society [IAS], Conference on Retroviruses and Opportunistic Infections [CROI], Infectious Diseases Society of America [IDSA], and the Inter Science Conference on Antimicrobial Agents and Chemotherapy [ICAAC]). Additionally, we reviewed bibliographies and contacted the authors for original data. We included abstracts if full-texts were not available.

Our search string (limited to humans) was: (1) “HIV”[MeSH] OR “HIV Seropositivity”[MeSH] OR “HIV Infections”[MeSH] AND (2) (“Self Care”[MeSH]) OR “Self Administration”[MeSH]) OR “Point-of-Care Systems”[MeSH] OR “self*test*” OR “rapid*test*.”

### Study Selection

**Figure 1:**
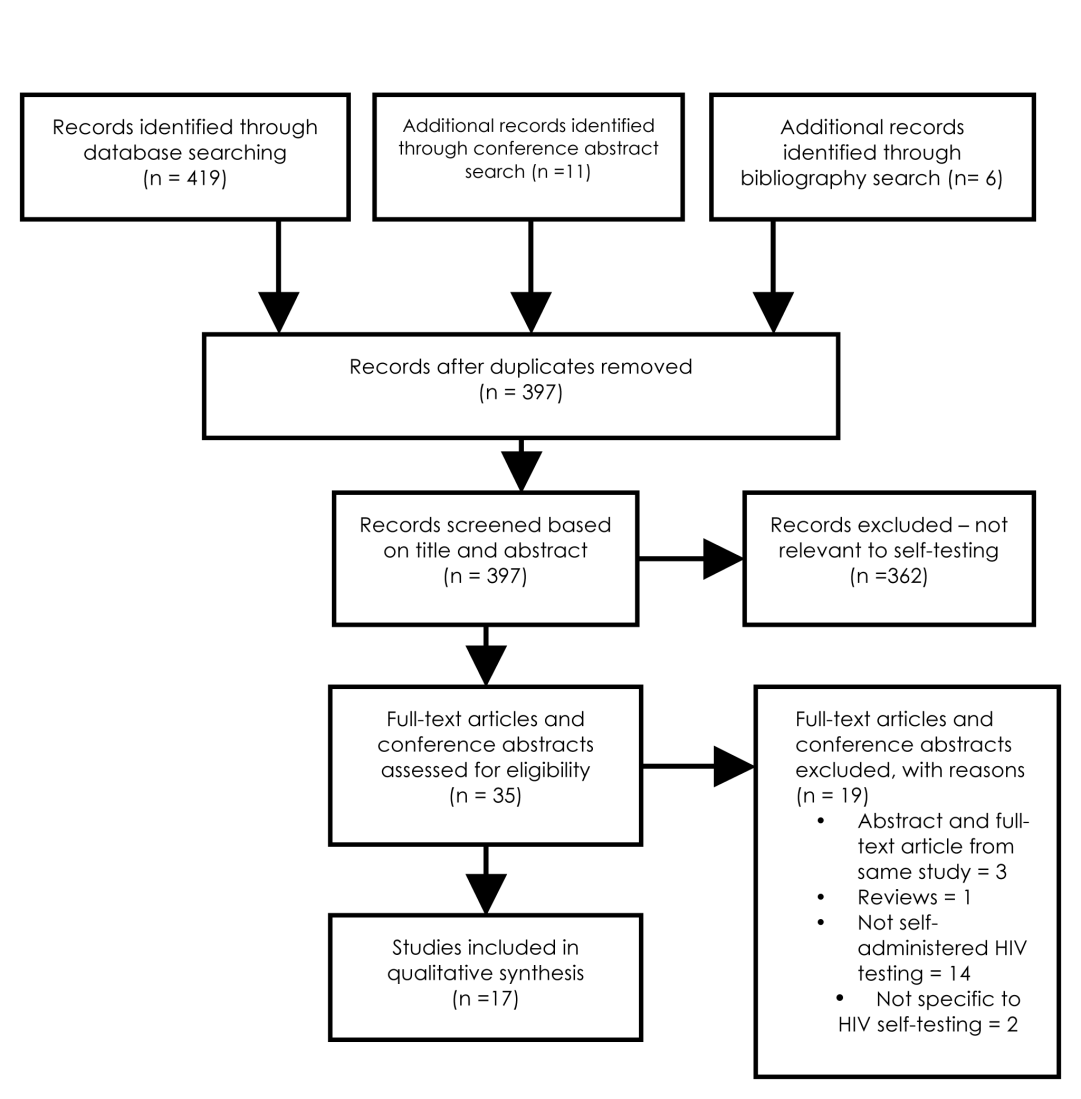
Flow chart of study search and selection.

Two reviewers (JS and SS) independently screened all citations. Please refer to the flow chart for details (Figure 1).

### Eligibility Criteria

Full-text articles, brief reports, or abstracts that evaluated HIV self-testing strategies in any part of the world were included. Reviews, perspectives, editorials, and studies that did not evaluate self-testing strategies (home-based non self test) were excluded (Figure 1).

### Data Abstraction

Two reviewers independently abstracted data from quantitative (JS and SS) and qualitative (SP and JS) studies. Concordance between reviewers was high at 90%. Disagreements were resolved by consultation with a third reviewer (NPP). A pre-piloted data abstraction form was used. Variables such as study characteristics, populations, study design, type of strategy, and outcomes were tabulated (Tables 2–4).

**Table 2:** Characteristics of included studies.

Test Strategy	Author Year	Study Setting	Sample Size	Survey Response Rate	HIV Self-Test Type	Study Design	Population	Summary Score for Quality Critique^a^
**Test strategy: supervised**								
	Skolnik 2001 [30]	USA	354	365/380	NA	Quantitative survey	HIV clinic attendees	56% (18/32)
	Spielberg 2003 (a) [32] b	USA	240	NA	Oral and finger-stick	Quantitative cross-sectional	HIV positive patients	55% (6/11)
	Spielberg 2003 (b) [31]	USA	460	460/865	NA	Quantitative survey	MSM. Persons from needle exchange site STI clinic attendees	63% (20/32)
	Lee 2007 [27]	Singapore	350	NA	Finger-stick	Quantitative cross-sectional	STI clinic attendees	69% (22/32)
	Spielberg 2007 [19] b	India	27	27	NA	Qualitative survey	General population, brought to community internet center	NA
	Chavula 2011 [17] b	Malawi	92	92	Oral (post-survey)	Qualitative survey	General urban population	NA
	Choko 2011 [33]	Malawi	283	NA	Oral	Quantitative cross-sectional	General urban population	72% (23/32)
	Gaydos 2011 [24]	USA	478	NA	Oral and finger-stick	Quantitative cross-sectional	Emergency department	59% (19/32)
	MacPherson 2011 [18]	Malawi	216	216/226	NA	Quantitative survey	General urban population	59% (19/32)
	Carballo-Dieguez 2012 (a) [16]	USA	57	NA	Oral	Qualitative and quantitative cross-sectional	Urban MSM	59% (19/32)
	Pant Pai 2012 [29] b	Canada	100	NA	Oral	Quantitative and qualitative cross-sectional	University students	82% (9/11)
	OraSure 2012 (phase IIb) [13]	USA	1,031	NA	Oral	Quantitative cohort study	Known HIV positives, general population	29% (10/34)
	Belza 2012 [21]	Spain	208	NA	Finger-stick	Quantitative cross-sectional	Attendees at a rapid HIV testing site	41% (13/32)
	Ng 2012 [28]	Singapore	994	NA	Oral	Quantitative cross-sectional	HIV positive, at-risk patients from family practice clinics	66% (21/32)
**Test strategy: unsupervised**								
	Kalibala 2011 [12]	Kenya	765	NA	Oral	Qualitative and quantitative cross-sectional	Health care professionals	28% (9/32)
	Katz 2012 [25] b	USA	108	NA	Oral	RCT	Urban MSM	59% (10/17)
	OraSure 2012 (phase III) [13]	USA	5,798	NA	Oral	Quantitative cohort study	General population high- (*n* = 4,689) and low- (*n* = 1,109) prevalence sites	29% (10/34)
	Fuente 2012 [23]	Spain	313^c^	NA	Finger-stick	Quantitative cross-sectional	Attendees at a rapid HIV testing site	56% (18/32)
	Lee 2012 [26] b	USA	500	NA	Oral	Quantitative cross-sectional	General population at unknown risk of HIV	64% (7/11)
	Carballo-Dieguez 2012 (b) [22] b	USA	28	NA	Oral	Qualitative and quantitative cross-sectional	HIV uninfected, urban non-monogamous MSM	55% (6/11)
	Helm 2012 [20] b	Netherlands	NA	NA	Oral	NA	NA	NA

a The summary score for quality critique represents the number of criteria reported, over the total number of criteria.

b Sample size for “cost preference and willingness to pay (WTP) (USD)” and “feasibility linkages errors” (Table 3) outcomes was 519, as data were reported in combination with participants from another testing program.

c Abstract.

NA, not available/not applicable; STI, sexually transmitted infection.

**Table 3:** Study outcomes: acceptability, accuracy, agreement and cost preference.

Test Strategy	Author Year	Study Setting	Acceptability	Accuracy	Agreement or Concordance	Cost Preference and WTP (US$)
**Test strategy: supervised**						
	**Skolnik 2001 ** [**30**]	USA	24%	NA	NA	NA
	**Spielberg 2003 (a) ** [**32**]	USA	NA	NA	Oral fluid test: 95%, blood-based test: 89%	70% WTP≤US$15; 40% WTP US$20
	**Spielberg 2003 (b) ** [**31**]	USA	NA	NA	NA	WTP US$10–US$15
	**Lee 2007 ** [**27**]	Singapore	NA	NA	κ value = 0.28 (*p*<0.01)	88% WTP US$7–US$13
	**Spielberg 2007 ** [**19**]	India	NA	NA	NA	NA
	**Gaydos 2011 ** [**24**]	USA	85%	NA	99.6%	NA
	**Carballo-Dieguez 2012 (a) ** [**16**]	USA	74%	NA	NA	NA
	**Pant Pai 2012 ** [**29**]	Canada	95%	NA	100%	Max WTP 20US$
	**OraSure 2012 (phase IIb) ** [**13**]	USA	NA	Sensitivity: 97.9% (95% CI 95.0–99.4); specificity: 99.79% (95% CI 98.1–100)	NA	NA
	**Belza 2012 ** [**21**]	Spain	78%	1% invalid	99% (95% CI 96.6–99.9)	NA
	**Ng 2012 ** [**28**]	Singapore	NA	Sensitivity: 97.4% (95% CI 95.1–99.7) Specificity: 99.9%(95% CI 99.6–100) 0.5% invalid	κ value = 0.97 (95% CI 0.95–0.99)	28% WTP>US$15
	**Chavula 2011 ** [**17**]	Malawi	NA	NA	NA	NA
	**Choko 2011 ** [**33**]	Malawi	92%	Sensitivity: 97.9% (95% CI 87.9–100) Specificity: 100% (95% CI 97.8–100)	NA	NA
	**MacPherson 2011 ** [**18**]	Malawi	92%	NA	NA	NA
**Test strategy: unsupervised**						
	**Kalibala 2011 ** [**12**]	Kenya	78%	NA	NA	NA
	**Katz 2012 ** [**25**]	USA	NA	NA	NA	45% WTP≤US$20; 25% WTP US$20–US$40, 17% WTP≥US$40, 13% WTP free
	**OraSure 2012 (phase III) ** [**13**]	USA	NA	**High-prevalence setting:** Sensitivity: 92.9% (95% CI 86.5–96.89); Specificity: 99.98% (95% CI 99.87–100); PPV: 99.1% (95% CI 94.86–99.98); NPV: 99.81% (95% CI 99.63–99.92) **Low-prevalence setting:** Sensitivity: 100% (95% CI NA; 0 FN); Specificity: 100% (95% CI 99.66–100); PPV: 100% (95% CI 2.5–100); NPV: 100% (95% CI 99.66–100)	NA	NA
	**Fuente 2012 ** [**23**]	Spain	NA	8% (95% CI 4.8–11.2) invalid tests	NA	18% WTP>US$38, 22% WTP US$25–US$38, 5.2% WTP free
	**Lee 2012 ** [**26**]	USA	NA	Specificity: 99.8% (95% CI 98.1–100), 1.8% testing error	NA	NA
	**Carballo-Dieguez 2012 (b) ** [**22**]	USA	84%	NA	NA	NA
	**Helm 2012 ** [**20**]	Netherlands	NA	NA	NA	NA

FN, false negative; K, kappa statistic; NA, not available; NPV, negative predictive value; PPV, positive predictive value; WTP, willingness to pay.

**Table 4:** Study outcomes: counselling preference, feasibility, linkages, errors, motivation, label comprehension, and test preference.

Test Strategy	Author Year	Study Setting	Counselling Preference	Feasibility, Linkages, Errors	Motivation, Label Comprehension	Test Preference
**Supervised self-testing strategies**						
	**Skolnik 2001 ** [**30**]	USA	100% for in-person pre and post test	NA	Convenience, speed, privacy, and anonymity	NA
	**Spielberg 2003 (a) ** [**32**]	USA	NA	Errors noted in placing test device in developer solution	NA	61% preferred testing at home
	**Spielberg 2003 (b) ** [**31**]	USA	NA	NA	NA	20% prefer home self-testing versus conventional test
	**Lee 2007 ** [**27**]	Singapore	79% for post-test counselling	NA	Convenience, speed, privacy, and anonymity	NA
	**Spielberg 2007 ** [**19**]	India	Computer-based pre and post test counselling	NA	Convenience, speed, privacy, and anonymity	NA
	**Gaydos 2011 ** [**24**]	USA	NA	5%–10% difficulties in test performance and test interpretation	NA	91% preferred oral fluid versus blood-based tests
	**Carballo-Dieguez 2012 (a) ** [**16**]	USA	NA	Errors in conduct: (1) touch test pad; (2) swab multiple times; (3) eating/drinking just before taking the test; (4) almost drinking the solution in the vial	87% for would likely self-test if available OTC and 80% would likely use it to test partners at home	NA
	**Pant Pai 2012 ** [**29**]	Canada	78% for post-test at community clinics, 53% post-test on the phone, 31% at pharmacies, 29% online	NA	98% convenience, 96% time efficient, 84% pain free	NA
	**OraSure 2012 (phase IIb) ** [**13**]	USA	NA	1.82% error rate in population of unknown status; 4.76% error in HIV positive population	NA	NA
	**Belza 2012 ** [**21**]	Spain	NA	1% invalid tests	NA	NA
	**Ng 2012 ** [**28**]	Singapore	72.5% for pre-test counselling; 73.9 for post-test counselling	Errors due to conduct: (1) use of collection pad to swap external lips; (2) touching the swab during removal from packaging; (3) spilling the test solutions; (4) misinterpret negative or invalid test results	Convenience, speed, privacy, and anonymity; kit instructions easy to understand	87.4% would but an OTC rapid test kit and 89% wanted to conduct HIV testing in private
	**Chavula 2011 ** [**17**]	Malawi	Post-test counselling considered essential	NA	NA	NA
	**Choko 2011 ** [**33**]	Malawi	90% preferred pre-test; 70% prefer in-person counselling over telephone counselling or information leaflets	Errors in conduct: (1) early removal of kit from the developer; (2) spilling the developer fluid	NA	NA
	**MacPherson 2011 ** [**18**]	Malawi	NA	NA	NA	NA
**Test strategy: unsupervised**						
	**Kalibala 2011 ** [**12**]	Kenya	Telephone-based counselling	NA		NA
	**Katz 2012 ** [**25**]	USA	NA		Convenience, speed, privacy, and anonymity	NA
	**OraSure 2012 (phase III) ** [**13**]	USA	88% sought post-test counselling	Test system failures: interpretational and operational errors: h**igh-prevalence settings:** 1.25% (95% CI 0.95%–1.63%); l**ow-prevalence settings:** 0.37% (95% CI 0.10%–0.93%); 96% newly diagnosed HIV positive subjects wanted to seek linkages and follow up;	97% would recommend oral self tests to others, 79% would use for self test	NA
	**Fuente 2012 ** [**23**]	Spain	NA	5.4% (95% CI 3.4–7.4) misinterpretation of self-test result picture; 6.6% with valid results find instructions “somewhat” or “quite difficult” versus 20% with invalid results		83.9% felt more motivated after taking the test to self-test in future
	**Lee 2012 ** [**26**]	USA	NA	NA	High-label comprehension among intended user populations 98.8% (95% CI 97.4–99.6)	NA
	**Carballo-Dieguez 2012 (b) ** [**22**]	USA	NA	NA	Availability of OTC would increase testing frequency	“High acceptability” among ethnic minority participants and ethnic minority sex partners
	**Helm 2012 ** [**20**]	Netherlands	NA	NA	NA	NA

NA, not available; WTP, willingness to pay.

### Quality Assessment and Data Synthesis

A quality critique of quantitative data from cross-sectional (Tables S1 and S2) and cohort studies (Table S3) was performed using the STROBE reporting checklist [11]. Two articles, although not peer reviewed, were critiqued using the STROBE checklist as they were reporting outcomes of cohort and cross-sectional studies [12],[13]. Similarly, a conference abstract reporting a RCT (Table S4) was appraised using the CONSORT guidelines [14]. A guide [15] for critically appraising qualitative research was used to appraise qualitative studies [12],[16]–[19]. The only study that could not be quality critiqued was an announcement of an implementation strategy through a conference abstract [20]. Due to lack of standardized reporting of primary and secondary outcomes, a meta-analysis was not conducted.

## Results

A total of 1,207 studies were identified from databases and bibliography searches and 14 abstracts retrieved from conferences for a total of 1,221 citations (please refer to Figure 1). After removing duplicates, 1,108 citations were reviewed in the first screen (Figure 1). Of 40 articles that were reviewed in the second screen, 20 were included. Reasons for exclusion of the remaining 20 studies were: repeat of reporting in abstract and full-text (*n* = 4), narrative review (*n* = 1), and topic irrelevant to this review (*n* = 15). One article reported on two separate studies, thus, a total of 21 studies were synthesized. Table 1 provides a list of definitions for the key outcomes or characteristics documented in this review. Table 2 presents the description of the 21 included studies, and Tables S1 to S4 present their detailed STROBE and CONSORT quality reporting assessment, where applicable.

**Table 1:** Definition of outcomes.

Outcome	Definition
**Acceptability**	Numerator defined as those individuals who chose to self-test.
	Denominator defined as all those who were offered and consented to test.
	Uptake = numerator/denominator (computed as a percentage)
**Accuracy**	Accuracy was defined by sensitivity and specificity parameters.
	Index test was a self-test result.
	Reference standard tests were combination of conventional lab tests for HIV (rapid tests or ELISA with p24 and/or Western blot depending on high- versus low-resource setting)
**Concordance**	Concordance for self-testing was reported as a measure of agreement between the test result between the individual and health care worker quantified either as percentage agreement or with the Cohen's Kappa (κ) inter-rater agreement
**Feasibility**	Documented completion of self-testing and counselling process.
	Includes ease of performance and interpretation of self-testing results, and documentation of errors, initiation of linkages.
**Motivators**	Factors contributing to the acceptability of HIV self tests

### Description of Included Studies

**Figure 2:**
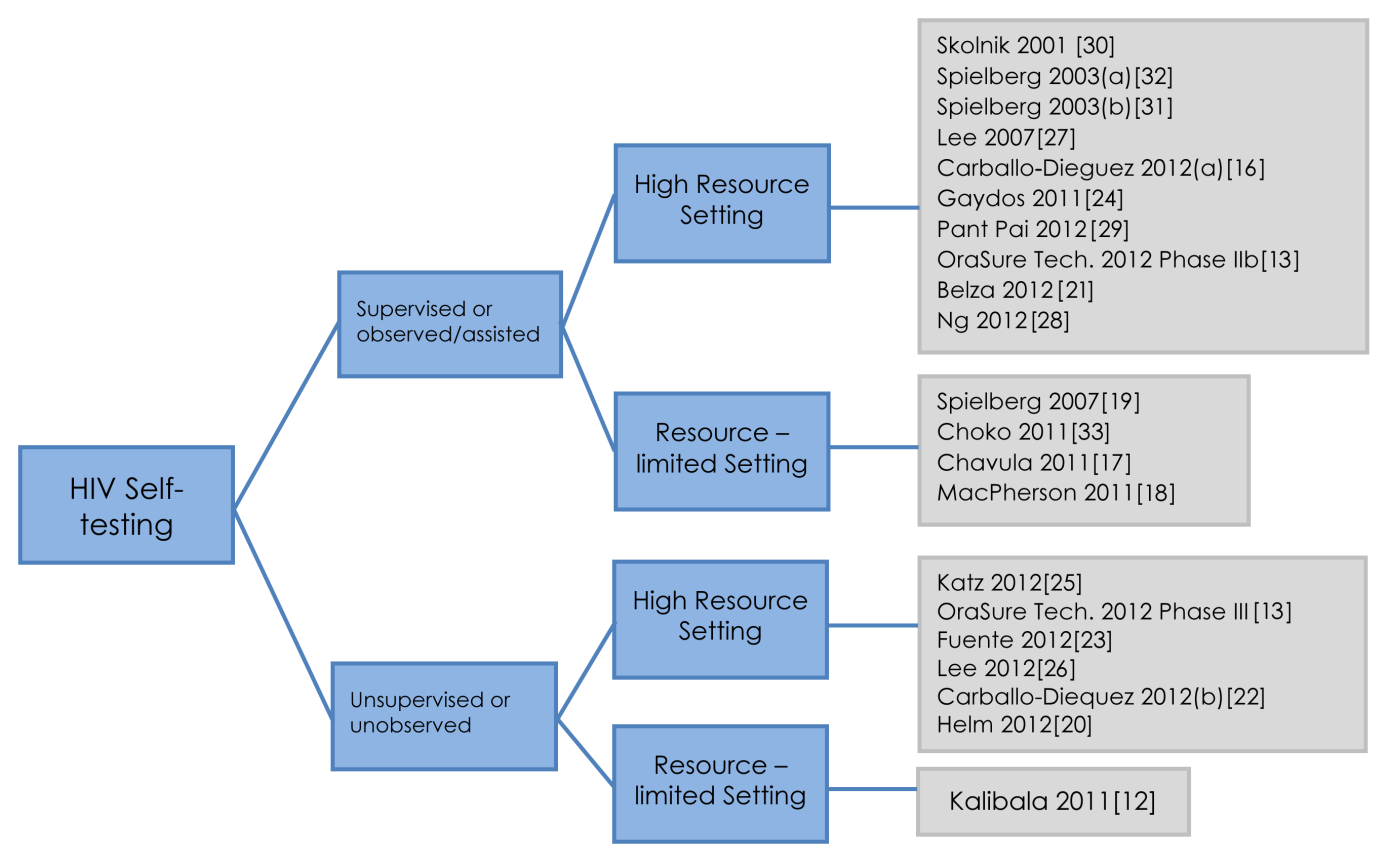
Self-testing strategies: a classification.

Of the 21 studies included, 16 (*n* = 16/21, 76%) were conducted in high-income [13],[16],[20]–[32] versus five (*n* = 5/21, 24%) in resource constrained country settings [12],[17]–[19],[33]. Total sample size varied from 27 to 5,798. Two main strategies for HIV self-testing were identified, supervised and unsupervised, and the studies classification is illustrated in Figure 2. The total sample size for the supervised testing strategy was 4,890 individuals and 7,512 for the unsupervised testing strategy. Our review provides data from 1,383 participants in resource constrained settings, compared to 11,019 in high-income settings, thus the bulk of data (89%) was from high-income settings. Study populations varied from high at-risk for HIV to low-risk general populations.

A vast majority of studies evaluated oral self tests (*n* = 14/21, 67%), while others used finger-stick-based tests. The two self-testing strategies, unsupervised and supervised, were evaluated with two oral POC tests, OraQuick and Calypte [12],[13],[16],[17],[20],[22],[24],[26]–[28],[29],[32],[33]. With the exception of one completed randomized controlled trial (RCT) [25], all other studies (*n* = 20/21, 95%) were observational, of which 14 (*n* = 14/20, 70%) were cross-sectional or cohort studies [12],[13],[16],[21]–[24],[26]–[29],[32],[33], while five (*n* = 5/20, 25%) were surveys [17]–[19],[30],[31], and one reported a study in progress that evaluated an unsupervised testing strategy coupled with Internet counselling for high-risk populations in the Netherlands [20].

A supervised self-testing strategy was evaluated in 14 (*n* = 14/21, 67%) studies, in both high- and low-risk populations with varying level of education, and access to resources. Although study participants performed the self test themselves, a HCP assisted the self-tester in any aspect of self-testing and counselling, if called upon. This assistance varied across studies, e.g., understanding the conduct of self-testing, helping with result interpretation, counselling, and initiating linkages for confirmatory testing.

In the unsupervised self-testing strategy (*n* = 7/21 studies, 33%), no assistance was offered by HCPs in the conduct and interpretation of self tests, but counselling was available off-site (on the phone or over the Internet). Unsupervised self-testing was evaluated primarily among urban and educated populations in high-income and resource constrained settings, such as a proposed evaluation in the Netherlands [20], HCPs in Kenya [12], urban men who have sex with men (MSM) in the US [25], general literate populations in the US [13], general population at unknown risk of HIV [26], urban non-monogamous MSM in the US [22], and attendees of a rapid HIV testing site in Spain [23].

### Quantitative Data

Table 1 provides the definitions used for reporting outcomes, facilitating their interpretation, documentation, and synthesis stratified by strategies.

### Acceptability

For both supervised and unsupervised self-testing strategies, acceptability (Table 3) was high (range: 74%–96%). For supervised strategies, acceptability was documented in seven studies. Acceptability varied from 74% (*n* = 42/57) in MSM in the US [16], to 78% (*n* = 208/267) in clinic attendees from Spain [21], to 85% (*n* = 478/564) in clinic attendees from the US [24], to 92% (*n* = 198/216 and *n* = 206/283) from household participants in Malawi [18],[33], to 95% (*n* = 100/105) in educated student populations in Canada [29], but was much lower (*n* = 85/354, 24%) in an early 2001 study in the US [30]. It should be noted that acceptability was measured in a research context, where participants voluntarily show up for self-testing. For unsupervised strategies, acceptability was documented in two studies, and ranged from 78% (*n* = 230/295) in HCPs in Kenya [12] to 84% (*n* = 27/32) in non-monogamous urban MSM in the US [22].

### Accuracy

Accuracy of self tests was reported by sensitivity and specificity parameters with the index self test being compared to the reference standard prevalent in each research setting. In high-resource country settings, the reference standard algorithm used was dual ELISA and Western blot performed on blood samples. In resource constrained settings, the three rapid test algorithm was employed.

Across both strategies, irrespective of the reference standard used, the specificity of the HIV self tests was consistently documented to be high, but sensitivity estimates varied greatly.

In the supervised testing strategy, high overall accuracy was generally observed. For example, a high sensitivity (97.9%; 95% CI 87.9–100) and a high specificity (100%; 95% CI 97.8–100) was reported in Malawi [33], as well as in a US-based study (sensitivity 97.9%; 95% CI 95.0–99.4—specificity 99.8%; 95% CI 98.1–100) [13], and in a Singapore-based study (sensitivity 97.4%; 95% CI 95.1–99.7—specificity 99.9%; 95% CI 99.6–100) [28]. However, in the unsupervised strategy, one study from the US reported a slightly lower sensitivity of 92.9% (95% CI 86.5–96.9) in a high-prevalence setting (HIV prevalence>1%), compared to a sensitivity of 100% (95% CI 99.7–100) in a low-prevalence setting (HIV prevalence≤1%) [13].

### Agreement and Concordance

Agreement and concordance between the self-tester and HCP could only be reported and computed for supervised testing strategies. Six studies reported data on concordance of self-testing results compared to test performed by HCPs [21],[24],[27]–[29],[32]. The five studies using oral self tests noted a higher agreement for oral self tests (95%–100% or κ = 0.97) [21],[24],[28],[29],[32] in comparison to finger-stick self tests (κ value = 0.28) [27]. Furthermore, a time trend was observed across studies, with a lower agreement reported in early studies (from 2001).

### Feasibility

Ten studies evaluated the feasibility of self-testing strategies, with various feasibility outcomes documented across studies. Feasibility outcomes included test conduct, test interpretation, post-test counselling, and treatment linkages.

In the unsupervised strategy, about 95% (*n* = 37/39) of urban MSM in the US found the kits to be “very easy to use” and no test errors were reported [25]. However, an implementation study evaluating the same strategy in the US in general populations reported on operator errors (interpretational and observational) [13]. These failures varied from 0.37% (*n* = 4/1,093) in low-prevalence settings to 1.25% (*n* = 56/4,465) in high-prevalence settings. A Spanish study evaluating an unsupervised strategy reported an overall interpretation error rate of 5.4% (*n* = 28/519) [23], which included a 1.1% (*n* = 11/1,038) error in interpretation of a positive result as negative.

Seven studies that evaluated supervised strategies documented errors in test performance [13],[16],[21],[24],[28],[32],[33]. Errors documented in general population in urban Malawi requested significantly more assistance from HCPs during self-testing with oral tests; errors were noted as a result of early removal of kit from the developer and spilling of the developer fluid [33]. Two studies from Singapore and Spain with finger-stick tests reported a high degree of errors, where 0.5% (*n* = 1/200) of participants obtained invalid self-test results [28] and about 1% (*n* = 2/208) of the finger-stick self-test results were found to be invalid [21]. In comparison, test conduct and performance errors noted with oral tests were: (i) collecting the oral mucosal sample, (ii) reading test instruction, and, (iii) interpreting test results [24].

In the study that evaluated both strategies, a higher error rate (4.76%, *n* = 24/504) was observed in test performance and interpretation by populations who tested positive for HIV in a supervised setting compared to unscreened or low-risk populations in an unsupervised strategy (1.25%, *n* = 56/4,465) [13]. This difference was attributed to the fact that, being already aware of their sero-status, individuals testing positive for HIV that self-tested did not care to read the instructions carefully and subsequently made more errors than unscreened populations. Two studies from the US have demonstrated that an improvement in self-test instructions for test conduct and interpretation reduce the incidence of errors [27],[32].

Data on post-test linkages were sparse. Linkage data were reported in only one study from the US that has evaluated an unsupervised strategy. It demonstrated that 96% (*n* = 102/106) of participants testing positive for HIV stated they would seek post-test counselling [13].

Overall in both strategies, a pattern of errors in self-test conduct was noted: (1) failing to place the oral test device in the developer solution after swabbing [32], (2) removing the test kit from the developer solution too early [33], (3) spilling the developer fluid [13],[28],[33], and lastly (4) dipping the test device in the developer solution before swabbing the gums [13],[16]. Likewise, errors in self-test interpretation were also reported: (1) difficulty in interpreting the test result on the device [24],[28] and (2) inability to read or interpret faint or weakly positive test lines on the test device [13].

### Motivators to Self-Test

Across all settings, both for the supervised and unsupervised self-testing strategies, motivators to self-testing were: (a) convenience, (b) speed and time to test result, (c) privacy, (d) a sense of empowerment, and, lastly, (e) a control of one's health choices [13],[16],[19],[22],[25]–[30].

In a supervised strategy evaluated in an educated population in Canada, convenience (99%, *n* = 99/100), time efficiency (97%, *n* = 97/100), and the pain-free procedure (84%, *n* = 84/100) were identified as motivators to self-testing [29]. In supervised strategies, in Malawi, participants preferred the distribution of test kits by one person (neighbourhood counsellor or trained person) and counselling by another person to maintain anonymity of self-testing [17]. Similarly, in a study with HIV clinic attendees in the US, 92% (*n* = 67/73) of participants cited provision of instant results, and 45% (*n* = 33/73) mentioned anonymity, confidentiality, and privacy as factors favoring self tests over conventional tests [30]. Likewise, in another supervised strategy evaluated in non-monogamous urban MSM in the US, 87% (*n* = 50/57) were likely to buy self tests, and 80% (*n* = 46/57) wanted to use it to test partners at home [16].

In unsupervised strategies, 7% (*n* = 19/288) of participants with valid results and 20% (*n* = 5/25) with invalid results found the test instructions “somewhat” or “quite” difficult to read, while most participants did not document difficulties following the test kit instructions [23]. High comprehension of instructions for use was also observed among intended user population in a study based in the US [26], and a study among urban non-monogamous MSM in the US documented that OTC availability of self tests would increase the frequency of testing in this population [22]. In another study evaluating an unsupervised testing strategy, a high percentage (79%, absolute number not reported) of HIV negative subjects expressed a desire to use a self test at home if available OTC, and 97% (absolute number not reported) of subjects newly diagnosed with HIV expressed a desire to recommend this OTC test to a friend [13].

### Preference

Three preference outcomes were documented: (a) preference for test and strategies, (b) cost preference and willingness to pay, and (c) preference for counselling.

#### Preference for tests and strategies

This outcome was variously documented. It included: (a) preference for oral versus finger-stick self test, (b) preference for self-testing strategy over conventional testing strategy, and (c) preference for self-testing of partners.

A majority (*n* = 14/21, 67%) of studies evaluated oral fluid tests for both strategies, and preference was attributed to the tests' non-invasiveness, convenience, and ease of specimen collection. Furthermore, in studies from the US where participants were offered a choice between oral and finger-stick, 91% (*n* = 433/478) chose oral fluid tests over blood-based tests (9%, *n* = 45/478) in one study [24], while in the other, 61% (*n* = 146/240) of participants preferred the oral test over the finger-stick test [32]. In a Canadian study with students, 81% (*n* = 81/100) preferred self tests over conventional lab tests, and 74% (*n* = 74/100) expressed a desire to buy them OTC [29].

For unsupervised strategies, in another US study in general populations and individuals of known positive HIV status, about 79% (absolute number not reported) preferred to self-test using oral over conventional tests, and 97% (absolute number not reported) wished to use oral tests to test their partners [13]. In Spain, 84% (*n* = 436/519) of the participants undergoing unsupervised testing felt more motivated after taking the test to self-test in the future [23]. Finally, a study among urban non-monogamous MSM in the US documented “high acceptability” of the unsupervised self-testing strategy among ethnic minority participants and their sex partners [22].

#### Cost preference and willingness to pay

Cost preference for self tests and willingness to pay if sold OTC were documented for both supervised and unsupervised strategies, and varied across populations, settings and strategies [23],[25],[27],[29]–[31],[32].

In supervised strategies, in Canada, 32% (*n* = 32/100) of university students were willing to pay up to US$10 and 41% (*n* = 41/100) up to US$20 [29], while in Singapore, 88% (*n* = 370/420) were willing to pay between US$7 and US$13, and 28% (*n* = 118/420) more than US$15 [27]. In a study in the US, 70% (*n* = 168/240) were willing to pay up to US$15, and 40% (*n* = 96/240) would be willing to pay US$20 for it [32]. In another study in the US, at-risk participants from homeless shelters wanted free self tests [30].

In unsupervised strategies evaluated in an urban MSM population, 45% (*n* = 49/108) were willing to pay less than US$20, 25% (*n* = 27/108) between 20 and US$40, 17% (*n* = 18/108) more than US$40 and 13% (*n* = 14/108) wanted it free [25]. Additionally, in Kenya, HCPs were unwilling to pay and wanted the government to provide it for free, as HIV was perceived to be an occupational risk [12]. A study in Spain evaluating an unsupervised strategy in attendees at a rapid HIV testing site documented 18% (*n* = 56/313) willingness to pay more than US$38, 22% (*n* = 69/313) between US$25 and US$28 and 5% (*n* = 16/313) wanting it for free [23].

#### Preference for counselling

Preferred mode and medium of counselling varied by strategies, as well as by educational and socio-economic status of the self-testers, which dictated their access to resources such as mobile phones, Internet, and pharmacies. In both supervised and unsupervised strategies, participants agreed that both pre- and post-counselling were essential components that needed to be integrated in the testing process [12],[13],[17],[19],[27]–[30],[33].

Regarding preference for counselling in supervised strategy, in two studies from Singapore, 79% and 74% (*n* = 275/350and *n* = 735/994) of participants felt that confidential post-test counselling was essential [27],[28]. In India, participants from the general population brought to community internet centers preferred computer-based counselling [19], while in San Francisco, homeless men preferred face-to-face counselling [30]. In a study from Malawi, participants indicated post-test counselling was essential, but agreed that it did not have to be immediately available following self-testing for test negative participants [17]. Regarding mode of counselling, in a study conducted in student populations in Montreal, students' preferred counselling options that were through community clinics (78%, *n* = 78/100), by phone (53%, *n* = 53/100), at pharmacies (31%, *n* = 31/100), and by Internet (29%, *n* = 29/100) [29]. In Malawi, most participants (including repeat testers) preferred face-to-face counselling over phone-based counselling [33].

Data for preference for and mode of counselling for the unsupervised strategy was limited. In the US, a high preference (88%, *n* = 93/106) was noted for post-test counselling, and 96% (*n* = 102/106) of subjects newly diagnosed with HIV indicated they would follow up for positive self-test results with a doctor or a clinic [13]. Among the subjects who tested positive for HIV who received post-test counselling, 69% (absolute number not reported) were calm upon learning their status, while 31% (absolute number not reported) indicated some level of anxiety; however, no intervention (suicide helpline) was required for any of these subjects [13]. In a study from Kenya, telephone-based counselling from a call-center was deemed useful by HCPs [12].

### Qualitative Data

Qualitative data were sparse and reported in five studies [12],[16]–[19]. A mixed methods study in Kenyan HCPs incorporated qualitative data to assess feasibility, acceptability, and barriers and the influence of self-testing on couples testing choices [12]. Another US-based study questioned MSM on the use of self tests to screen potential sexual partners [16]. A study in urban India surveyed general population at community internet centers [19], while two other US-based studies elucidated social and structural barriers (challenges, concerns, and barriers) [17],[18]; these issues are discussed in detail below.

### Challenges

Challenges in self-testing with a partner were discussed for both strategies: unsupervised strategy among HCPs in Kenya and supervised strategy in high-risk MSM in the US [12],[16]. Kenyan HCPs noted that the key challenge was avoiding the potential misuse of self tests, including non consensual testing [12]. In another study that evaluated a supervised strategy with non-monogamous MSM, refusal of new partners to self-test was flagged as a possible positive HIV status that would deter them from having sex or encourage them to use condoms [16]. Participants also indicated that the self test would “kill the mood” if used right before sex and being under the influence of drugs and/or alcohol could also dissuade testing altogether [16].

### Concerns

Concerns regarding both strategies included accuracy, stigma, misuse, and potential abuse of self tests. A lack of trust in the accuracy of self-test results was reported in two studies [29],[30], while in one US-based study, 94% (*n* = 407/433) of participants trusted their oral fluid results more than their finger-stick results (87%, *n* = 39/45) [24]. In supervised self-testing strategies, where part of the self-test distribution or assistance was offered by counsellors or provided at visible testing centers, perceived stigma as a barrier was reported by participants in two studies [18],[19]. HCPs in Kenya described potential misuse of self tests including non-consensual testing of partners and children/infants, maliciously infecting others if found positive, and unauthorized selling of the test kit [12].

### Barriers

Study participants for both strategies in both settings perceived barriers. In the supervised testing strategy, stigma and discrimination, and sequelae surrounding a diagnosis of HIV was high in Malawi, with 22% (*n* = 47/216) of participants fearing verbal abuse, 14% (*n* = 29/216) thinking they would be excluded by friends, and 10% (*n* = 11/110) of women and 11.3% (*n* = 12/106) of men fearing that their partner would leave them in case they tested positive for HIV [18]. In an unsupervised strategy evaluated in Kenya, a fear of visibility discouraged HCPs because they did not want to be seen carrying the self-test kits [12].

### Quality of Studies

Quality of studies varied. A lack of standardization in reporting and documentation of outcomes was observed. Qualitative data were sparse and evaluation of quality limited by incomplete reporting of data in abstracts. Combined reporting of quantitative and qualitative findings along with insufficient reporting of the qualitative methods and data collection tools (e.g., content of interview guide) may have masked potentially useful qualitative evidence. In a US study, the rationale for employment of different methodologies was clearly explained, and care was taken to reduce bias [16]. However, lack of clear presentation of themes in the results limited our understanding of collected data.

## Discussion

We identified 21 studies that assessed supervised or unsupervised HIV self-testing strategies. The majority of the evidence from included studies was for supervised strategies from high-resource settings, although seven recent studies reported on an unsupervised strategy [12],[13],[20],[22],[23],[25],[26]. Included studies recorded a high score for acceptability (range: 74%–96%), preference over facility-based testing (range: 61%–91%), and partner self-testing (range: 80%–97%). Sensitivity and specificity for both unsupervised and supervised self-test strategies was high, but a lower sensitivity was reported for unsupervised (range: 92.9%–100%; one study) versus supervised (range: 97.4%–97.9%; three studies) strategies. Only one of the included studies reported on linkage to counselling and care where 96% (*n* = 102/106) of individuals who tested positive for HIV stated they would seek post-test counselling.

While the evidence of high acceptability for supervised strategies is clear, it is not so for unsupervised strategies, especially in resource constrained settings. Only one study evaluated it, and the study focused on acceptability in a health literate population (HCPs) [12]. In terms of interpretation, self-testing has to be viewed as a process that requires a higher level of motivation and pro-activity compared to facility-based conventional testing. It involves a certain level of independence and assumes a basic level of literacy (typically grade 6 or high school) to ensure confidence in the self-test conduct and interpretation and enough personal involvement to seek post-test counselling and linkage to care and treatment. In addition to motivation, the conduct of a self test and its interpretation are key to its successful implementation. Additionally, demystifying the process of self-testing on television and social media will improve passive absorption, help de-stigmatize the HIV testing process, and promote the visibility of a self-testing strategy while providing information on HIV. Therefore, more research on exploring the best strategy (i.e., supervised versus unsupervised strategies) for different high- and low-risk populations in resource-constrained settings is clearly needed.

Evidence for preference for self tests was evident for both supervised and unsupervised strategies, while mode and medium of preferences for counselling varied across settings, populations, and strategies. In terms of interpretation, qualitative research will help understand and tailor the preferred counselling option to high- and low-risk populations in diverse settings. Counselling strategies must be tailored to the literacy levels, lifestyle needs, and preferences, especially for populations living in resource constrained settings.

For cost preferences, low-income populations in both high- and low-resource settings tended to prefer free self tests whereas higher income groups in both settings were willing to pay typically up to US$20. This finding implies that the price of a self-test kit will be an important factor in determining the uptake of self tests. Qualitative research will help guide the ranges of cost preference. This is crucial to policy initiatives for both strategies in both settings.

Regarding findings on accuracy, while a consistently high specificity was noted for both strategies across all settings, a wide variability in sensitivity estimates was observed. Lower sensitivity was more prominent in studies that used the unsupervised self-testing strategy. Interestingly, lower estimates of sensitivity were also reported from high-prevalence settings, conducted by at-risk testers that were experienced with testing (including individuals who knew their sero-status) but generated more errors with the self test by not complying with instructions compared to individuals who did not know their sero-status. In contrast, naïve self-testers in low-risk settings seemed to follow instructions diligently resulting in high estimates of sensitivity. Although this process of passive absorption and priming to self-test instructions was present in supervised strategies in high-income settings where it resulted in fewer errors and highly accurate results, it was notably absent or weakly implemented in the unsupervised strategy. This finding highlights the importance of improving clarity of instructions to self-test. In terms of generalizability, adapting these instructions to contexts and populations with use of various media (pictorial and textual instructions, web, social media, or smartphone-based content in a user friendly format) and tailoring to languages and levels of literacy especially for low-resource settings will be essential.

In terms of feasibility, none of the existing literature addressed issues related to seeking linkage to care (obtaining a CD4 count or having visited a clinic for ART initiation) and evidence from only one study reported that 96% (*n* = 102/106) of individuals who tested positive for HIV stated they would seek post-test counselling. Linkages could be better documented in the next phase of controlled studies.

Furthermore, in planning roll out of self-testing strategies, clarification of some concepts and limitations of the self tests to potential self-testers is necessary. These include presence of faint positive lines and their interpretation as a positive test result, and the limitation of antibody-based self tests to detect an HIV infection only after 90 d. With either strategy, occurrence of false negatives or indeterminate test outcomes cannot be eliminated with antibody-based oral or finger-stick self tests so repeat testing at 3-mo intervals should always be encouraged. At any time, calling or meeting a counsellor for clarification of test results must be emphasized.

For poor and less literate populations who cannot afford self tests or cannot comprehend the process of testing, the supervised self-testing strategy may remain the best option. But this option requires a careful controlled evaluation in many sub-Saharan and Asian settings. Engagement of providers and stakeholders is a necessary first step before implementation of a self-testing strategy because treatment and staging must be available for newly diagnosed individuals with HIV. Efforts must be made to avoid loss to follow up.

In under regulated and under funded health systems, it will be important to regulate the quality of self-test kits along with the process of self-testing. In contrast, in well-managed health systems in high-income countries (e.g., US, Canada, UK, Australia, Singapore, Japan) and populations with high socio-economic status in emerging economies and middle-income countries (China, South Africa, Russia, India, Brazil, Colombia), affordable OTC self tests sold at pharmacies or online should work well with linkages operationalized by counsellors via different mediums, such as over the phone, the Internet, or face-to-face at a clinic.

Evidence of extreme adverse effects, for example suicide, and the potential for abuse of self tests was consistently absent. However, as such evidence is collected in well-controlled studies, such data from a real world context will be hard to document. Anecdotal evidence suggests that unregulated rapid tests are available in markets of African and Asian countries and that such abuse will happen regardless of the introduction of self tests. Nevertheless, safeguards introduced by public health systems could help minimize the problem. To reach preliminary self-testers, a 24/7 toll free number to reach counsellors in time, and the offer of an option of face-to-face counselling could help obviate the occurrence.

In terms of new research, controlled clinical trials in different populations and settings with exploration of tailored and novel linkage modalities are urgently needed. This will generate the best evidence on offering an optimal and tailored strategy suited to the needs of different populations and settings. The impact of self-testing on detecting new cases of HIV in the community and changes in sexual behaviour of self-testers post-testing are yet to be assessed. Because data on prior HIV testing behaviour for unsupervised strategies were undocumented, new knowledge on whether self-testing reached new testers is unclear and therefore needed. Data on the impact of self-testing in increasing community engagement in screening, and on long term HIV outcomes remain limited. These data can be documented through community-based cluster RCTs or implementation research. However, it is hard to conduct such trials in communities when self tests are not approved for use in many countries and especially in health systems where linkage to counselling is not in place. Lastly, formative research that further explores individual motivations in using self tests, their fears, perceptions, and concerns, is needed. It will assist in tailoring self-testing to the needs and preferences of individuals and communities.

In sum, self-testing offers an alternative for individuals who desire privacy and confidentiality to find out their sero-status, as well as for under-resourced health care systems and settings where stigma and discrimination may prevail. It offers the potential to bring more people to self-screen and proactively seek linkage to care and prevention, but its potential in optimizing linkages remains unproven. However, if optimized, self-testing could facilitate and motivate individuals who preliminarily tested positive for HIV to seek care and counselling. Furthermore, care seeking should not be left to the motivated self-tester, but facilitated through various means, including technological innovations. But in any such system, if any component of self-testing, linked counselling and initiation of care are poorly managed, then the self-testing strategy could cause more harm than good. At all times therefore, linking positive self-test results within an 8–24-h window period to post-test counselling followed up by CD4 count and ART initiation should continue to be emphasized.

### Strengths and Limitations

Approximately 70% of the included studies were cross-sectional in design. The majority of the data were primarily derived from implementation studies and from high-income settings (89%), only one of which was an RCT. Few studies were conducted in resource constrained countries, none of which were RCTs. Lack of data from resource constrained settings limited our comparisons of supervised versus unsupervised test strategies (one unsupervised study of HCPs in Kenya [12] and one supervised study of an urban population in Malawi [33]). Data on linkage to care was sparse with only one unsupervised self-testing study reporting linkage to care as an outcome. Selection and volunteer bias (self-selection of participants) within included studies cannot be ruled out. Publication and reporting bias are also likely. Errors in test interpretation were recorded in three studies. With these errors, a potential misclassification of false negative test results and misinterpretation of faint positive lines is probable; hence information bias (misclassification of test results) is also likely.

The quality of reporting in included studies was generally poor. STARD and QUADAS checklists could not be used to assess quality because they are tailored for diagnostic accuracy, which was not the primary outcome in most studies. The use of observational study designs, data from pilot studies, with incomplete reporting of data items and lack of compliance with STROBE reporting criteria, were typical problems encountered in our quality assessment of the studies. A general lack of standardized reporting of outcomes beyond accuracy (patient centered outcome or implementation research outcomes) in the field of POC diagnostics has been noted [34]. It is therefore not surprising that these outcomes were heterogeneously and incompletely reported across many studies. The wide range of sources searched, the lack of language restrictions, and the evaluation of study quality are strengths of this review.

### Conclusion

Privacy, anonymity, time-savings, and convenience facilitated a high acceptability and preference for both supervised and unsupervised self-testing strategies across diverse global settings. Included studies demonstrated that it was feasible to implement both supervised and unsupervised oral fluid-based self-testing strategies, despite variable accuracy estimates obtained by self-testers. However data on linkages to care were only reported by a single study of unsupervised self-testing. Self-testing can be an alternative option to facility-based testing for individuals who desire more privacy. However, controlled trials wherever possible, and implementation studies that document linkage to care, are warranted to confirm findings from observational studies. Although self-testing offers the potential to increase the number of individuals to self-screen for HIV and therefore deliver more people to care, systems that can maintain confidentiality and operationalize linkage to care within a reasonable time frame are pertinent to its success. More data from diverse settings are needed to inform global scale-up and policy recommendations for HIV self tests.

## Supporting Information

Table S1 STROBE reporting criteria for cross-sectional studies (full-text).
(DOCX)

Table S2 STROBE reporting criteria for cross-sectional studies (conference abstracts).
(DOCX)

Table S3 STROBE reporting criteria for cohort study (full-text).
(DOCX)

Table S4 CONSORT reporting criteria for RCT (conference abstract).
(DOCX)

Text S1 PRISMA checklist.
(DOC)

## Abbreviations

HCP: health care professional
MSM: men who have sex with men
OTC: over the counter
POC: point of care
RCT: randomized controlled trial
