# Supplementary material for: Supervised and Unsupervised Self-Testing for HIV in High- and Low-Risk Populations: A Systematic Review
Source: PLoS Med. 2013 Apr 2;10(4):e1001414. doi: 10.1371/journal.pmed.1001414 (PMC3614510; doi:10.1371/journal.pmed.1001414)
Supplement: Table S1 — STROBE reporting criteria for cross-sectional studies (full-text). (DOCX) [file pmed.1001414.s001.docx]

**Table S1: STROBE reporting criteria for cross-sectional studies (full-text)**

| **STROBE Recommendation**  ***(For cross-sectional studies – full-text)*** | **Skolnik 2001[32]** | **Spielberg 2003 (b)[33]** | **Lee 2007[29]** | **Choko 2011[35]** | **Gaydos 2011[26]** | **Kalibala 2011[14]** | **MacPherson 2011[20]** | **Carballo-Dieguez 2012 (a)[18]** | **Belza 2012[23]** | **Ng 2012[30]** | **Fuente 2012[25]** |
| --- | --- | --- | --- | --- | --- | --- | --- | --- | --- | --- | --- |
| 1. **Indicate the study’s design with a commonly used term in the title or the abstract** | NR | R | R | R | R | NR | R | R | R | R | NR |
| 1. **Provide in the abstract an informative and balanced summary of what was done and what was found** | R | R | R | R | R | R | R | R | R | R | R |
| 1. **Explain the scientific background and rationale for the investigation being reported** | R | R | R | R | NR | R | R | R | R | R | R |
| 1. **State specific objectives, including any pre-specified hypotheses** | R | R | R | R | R | R | R | R | R | R | R |
| 1. **Present key elements of study design early in the paper** | R | R | R | R | R | R | R | R | R | R | R |
| 1. **Describe the setting, locations, and relevant dates, including periods of recruitment, exposure, follow-up, and data collection** | R | R | R | R | R | R | R | R | R | R | R |
| 1. **Give the eligibility criteria, and the sources and methods of selection of participants** | R | R | R | R | R | R | R | R | R | R | R |
| 1. **Clearly define all outcomes, exposures, predictors, potential confounders, and effect modifiers. Give diagnostic criteria, if applicable** | R | R | R | R | R | NR | R | R | R | R | R |
| 1. **For each variable of interest, give sources of data and details of methods of assessment (measurement). Describe comparability of assessment methods if there is more than one group** | R | R | R | R | R | NR | R | R | R | R | R |
| 1. **Describe any efforts to address potential sources of bias** | NR | NR | NR | NR | NR | NR | NR | NR | NR | R | NR |
| 1. **Explain how the study size was arrived at** | R | R | NR | R | R | NR | NR | NR | NR | NR | NR |
| 1. **Explain how quantitative variables were handled in the analyses. If applicable, describe which groupings were chosen and why** | NR | R | R | R | R | NR | R | R | NR | R | R |
| 1. **Describe all statistical methods, including those used to control for confounding** | NR | R | R | R | NR | NR | NR | R | NR | R | NR |
| 1. **Describe any methods used to examine subgroups and interactions** | NR | NR | R | R | NR | NR | NR | NR | NR | NR | NR |
| 1. **Explain how missing data were addressed** | NR | NR | NR | NR | NR | NR | NR | NR | NR | NR | NR |
| 1. **If applicable, describe analytical methods taking account of sampling strategy** | NR | NR | NR | R | NR | NR | NR | NR | NR | NR | NR |
| 1. **Describe any sensitivity analyses** | NR | NR | NR | NR | NR | NR | NR | NR | NR | NR | NR |
| 1. **Report numbers of individuals at each stage of study—eg numbers potentially eligible, examined for eligibility, confirmed eligible, included in the study, completing follow-up, and analyzed** | R | R | R | R | R | NR | R | R | NR | R | NR |
| 1. **Give reasons for non-participation at each stage** | NR | NR | R | NR | NR | NR | NR | R | NR | NR | NR |
| 1. **Consider use of a flow diagram** | NR | NR | NR | NR | NR | NR | NR | NR | NR | NR | R |
| 1. **Give characteristics of study participants (e.g. demographic, clinical, social) and information on exposures and potential confounders** | R | R | R | R | R | R | R | R | R | R | R |
| 1. **Indicate number of participants with missing data for each variable of interest** | NR | NR | NR | R | NR | NR | NR | NR | NR | NR | R |
| 1. **Report numbers of outcome events or summary measures** | R | R | R | R | R | R | R | NR | R | R | R |
| 1. **Give unadjusted estimates and, if applicable, confounder-adjusted estimates and their precision (e.g., 95% confidence interval). Make clear which confounders were adjusted for and why they were included** | R | NR | R | R | NR | NR | R | NR | NR | R | R |
| 1. **Report category boundaries when continuous variables were categorized** | NR | NR | NR | NR | NR | NR | NR | NR | NR | NR | NR |
| 1. **If relevant, consider translating estimates of relative risk into absolute risk for a meaningful time period** | NR | NR | NR | NR | NR | NR | NR | NR | NR | NR | NR |
| 1. **Report other analyses done—eg analyses of subgroups and interactions, and sensitivity analyses** | NR | NR | NR | NR | R | NR | NR | NR | NR | NR | NR |
| 1. **Summarize key results with reference to study objectives** | R | R | R | R | R | R | R | R | R | R | R |
| 1. **Discuss limitations of the study, taking into account sources of potential bias or imprecision. Discuss both direction and magnitude of any potential bias** | R | R | R | R | R | NR | R | R | NR | R | R |
| 1. **Give a cautious overall interpretation of results considering objectives, limitations, multiplicity of analyses, results from similar studies, and other relevant evidence** | R | R | R | R | R | NR | R | R | NR | R | R |
| 1. **Discuss the generalizability (external validity) of the study results** | R | R | R | NR | R | NR | R | R | NR | R | NR |
| 1. **Give the source of funding and the role of the funders for the present study and, if applicable, for the original study on which the present article is based** | R | R | R | R | R | NR | R | R | R | R | R |

R-reported, NR not reported.
