# Supplementary material for: Supervised and Unsupervised Self-Testing for HIV in High- and Low-Risk Populations: A Systematic Review
Source: PLoS Med. 2013 Apr 2;10(4):e1001414. doi: 10.1371/journal.pmed.1001414 (PMC3614510; doi:10.1371/journal.pmed.1001414)
Supplement: Table S2 — STROBE reporting criteria for cross-sectional studies (conference abstracts). (DOCX) [file pmed.1001414.s002.docx]

**Table S2: STROBE reporting criteria for cross-sectional studies (conference abstracts)**

| **STROBE Recommendations**  **(*For conference abstracts)*** | **Spielberg 2003 (a)[34]** | **Pant Pai 2012[31]** | **Lee 2012[28]** | **Carballo-Dieguez 2012 (b)[24]** |
| --- | --- | --- | --- | --- |
| 1. **Indicate the study’s design with a commonly used term in the title** | NR | R | NR | NR |
| 1. **Contact details for the corresponding author** | R | R | R | R |
| 1. **Description of the study design** | NR | R | NR | NR |
| 1. **Specific objectives or hypothesis** | R | R | R | R |
| 1. **Description of setting, follow-up dates or dates at which the outcome events occurred or at which the outcomes were present, as well as any points or ranges on other time scales for the outcomes** | R | R | R | R |
| 1. **Give the eligibility criteria, and the major sources and methods of selection of participants** | NR | R | R | R |
| 1. **Clearly define primary outcome for this report.** | R | R | R | NR |
| 1. **Describe statistical methods, including those used to control for confounding** | NR | NR | NR | NR |
| 1. **Report Number of participants at the beginning and end of the study** | R | R | R | R |
| 1. **Report estimates of associations. If relevant, consider translating estimates of relative risk into absolute risk for a meaningful time period Report appropriate measures of variability and uncertainty (e.g., odds ratios with confidence intervals** | NR | NR | NR | NR |
| 1. **General interpretation of study results** | R | R | R | R |

R-reported, NR not reported.
