# Supplementary material for: Supervised and Unsupervised Self-Testing for HIV in High- and Low-Risk Populations: A Systematic Review
Source: PLoS Med. 2013 Apr 2;10(4):e1001414. doi: 10.1371/journal.pmed.1001414 (PMC3614510; doi:10.1371/journal.pmed.1001414)
Supplement: Table S3 — STROBE reporting criteria for cohort study (full-text). (DOCX) [file pmed.1001414.s003.docx]

**Table S3: STROBE reporting criteria for cohort study (full-text)**

| **STROBE Recommendations**  **(*For full-text)*** | **OraSure 2012 (Phase IIb and III)[15]** |
| --- | --- |
| 1. **Indicate the study’s design with a commonly used term in the title or the abstract** | NR |
| 1. **Provide in the abstract an informative and balanced summary of what was done and what was found** | NR |
| 1. **Explain the scientific background and rationale for the investigation being reported** | NR |
| 1. **State specific objectives, including any prespecified hypotheses** | R |
| 1. **Present key elements of study design early in the paper** | R |
| 1. **Describe the setting, locations, and relevant dates, including periods of recruitment, exposure, follow-up, and data collection** | R |
| 1. **Give the eligibility criteria, and the sources and methods of selection of participants. Describe methods of follow-up** | R |
| 1. **For matched studies, give matching criteria and number of exposed and unexposed** | NR |
| 1. **Clearly define all outcomes, exposures, predictors, potential confounders, and effect modifiers. Give diagnostic criteria, if applicable** | NR |
| 1. **For each variable of interest, give sources of data and details of methods of assessment (measurement). Describe comparability of assessment methods if there is more than one group** | NR |
| 1. **Describe any efforts to address potential sources of bias** | NR |
| 1. **Explain how the study size was arrived at** | NR |
| 1. **Explain how quantitative variables were handled in the analyses. If applicable, describe which groupings were chosen and why** | NR |
| 1. **Describe all statistical methods, including those used to control for confounding** | NR |
| 1. **Describe any methods used to examine subgroups and interactions** | NR |
| 1. **Explain how missing data were addressed** | NR |
| 1. **If applicable, explain how loss to follow-up was addressed** | NR |
| 1. **Describe any sensitivity analyses** | NR |
| 1. **Report numbers of individuals at each stage of study—eg numbers potentially eligible, examined for eligibility, confirmed eligible, included in the study, completing follow-up, and analyzed** | R |
| 1. **Give reasons for non-participation at each stage** | NR |
| 1. **Consider use of a flow diagram** | R |
| 1. **Give characteristics of study participants (eg demographic, clinical, social) and information on exposures and potential confounders** | R |
| 1. **Indicate number of participants with missing data for each variable of interest** | NR |
| 1. **Summarize follow-up time (eg, average and total amount)** | NR |
| 1. **Report numbers of outcome events or summary measures over time** | R |
| 1. **Give unadjusted estimates and, if applicable, confounder-adjusted estimates and their precision (eg, 95% confidence interval). Make clear which confounders were adjusted for and why they were included** | NR |
| 1. **Report category boundaries when continuous variables were categorized** | NR |
| 1. **If relevant, consider translating estimates of relative risk into absolute risk for a meaningful time period** | NR |
| 1. **Report other analyses done—eg analyses of subgroups and interactions, and sensitivity analyses** | NR |
| 1. **Summarize key results with reference to study objectives** | R |
| 1. **Discuss limitations of the study, taking into account sources of potential bias or imprecision. Discuss both direction and magnitude of any potential bias** | NR |
| 1. **Give a cautious overall interpretation of results considering objectives, limitations, multiplicity of analyses, results from similar studies, and other relevant evidence** | NR |
| 1. **Discuss the generalizability (external validity) of the study results** | NR |
| 1. **Give the source of funding and the role of the funders for the present study and, if applicable, for the original study on which the present article is based** | R |

R-reported, NR not reported.
