# Supplementary material for: Supervised and Unsupervised Self-Testing for HIV in High- and Low-Risk Populations: A Systematic Review
Source: PLoS Med. 2013 Apr 2;10(4):e1001414. doi: 10.1371/journal.pmed.1001414 (PMC3614510; doi:10.1371/journal.pmed.1001414)
Supplement: Table S4 — CONSORT reporting criteria for RCT (conference abstract). (DOCX) [file pmed.1001414.s004.docx]

**Table S4: CONSORT reporting criteria for RCT (conference abstract)**

| **CONSORT Recommendations**  **(*For conference abstracts)*** | **Katz 2012[27]** |
| --- | --- |
| 1. **Identification of the study as randomized in the title** | NR |
| 1. **Contact details for the corresponding author** | R |
| 1. **Description of the trial design (e.g. parallel, cluster, non-inferiority)** | R |
| 1. **Eligibility criteria for participants and the settings where the data were collected** | R |
| 1. **Interventions intended for each group** | R |
| 1. **Specific objective or hypothesis** | R |
| 1. **Clearly defined primary outcome for this report** | R |
| 1. **How participants were allocated to interventions** | NR |
| 1. **Whether or not participants, care givers, and those assessing the outcomes were blinded to group assignment** | R |
| 1. **Number of participants randomized to each group** | NR |
| 1. **Trial status** | NR |
| 1. **Number of participants analysed in each group** | NR |
| 1. **For the primary outcome, a result for each group and the estimated effect size and its precision** | R |
| 1. **Important adverse events or side effects** | R |
| 1. **General interpretation of the results** | R |
| 1. **Registration number and name of trial register** | NR |
| 1. **Source of funding** | NR |

R-reported, NR not reported.
